# Pre-Infection Nutritional Status, Oxidative Stress, and One-Year-Long COVID Persistence in Patients Undergoing Hemodialysis: A Prospective Cohort Study

**DOI:** 10.3390/clinpract14030070

**Published:** 2024-05-17

**Authors:** Natalia Stepanova, Lesya Korol, Tetyana Ostapenko, Valeriia Marchenko, Olga Belousova, Lyudmyla Snisar, Iryna Shifris, Mykola Kolesnyk

**Affiliations:** 1 State Institution “O.O. Shalimov National Scientific Center of Surgery and Transplantology of the National Academy of Medical Science of Ukraine”, 03680 Kyiv, Ukraine; 2 Dialysis Medical Center LLC “Nephrocenter”, 03057 Kyiv, Ukraine; 3 Dialysis Medical Center LLC “Nephrocenter”, 69035 Zaporizhzhia, Ukraine

**Keywords:** long COVID, hemodialysis, controlling nutritional status score, undernutrition, oxidative stress, persistence

## Abstract

Background: Nutritional status’s role in long COVID is evident in the general population, yet unexplored in patients undergoing hemodialysis (HD), posing a research gap. We hypothesized that pre-infection undernutrition in HD patients might impact long COVID persistence by accelerating oxidative stress. The present study aimed to investigate the association between pre-infection nutritional status, oxidative stress, and one-year-long COVID persistence in HD patients. Methods: This prospective observational cohort study enrolled 115 HD patients with confirmed COVID-19. Nutritional status was assessed using the Controlling Nutritional Status (CONUT) score twice: before infection and three months post-infection. Oxidative markers included malondialdehyde (MDAs), ceruloplasmin, transferrin, and sulfhydryl groups. The endpoint was one-year-long COVID persistence. Results: Moderate pre-infection CONUT scores were associated with heightened severe undernutrition risk (*p* < 0.0001), elevated MDAs (*p* < 0.0001), and reduced ceruloplasmin levels (*p* = 0.0009) at three months post-COVID-19 compared to light CONUT scores. Pre-infection CONUT score independently predicted post-COVID oxidative damage [OR 2.3 (95% CI 1.2; 4.6), *p* < 0.0001] and one-year-long COVID persistence [HR 4.6 (95% CI 1.4; 9.9), *p* < 0.0001], even after adjusting for potential confounders. Conclusion: Moderate pre-infection undernutrition heightens post-COVID oxidative stress and increases the risk of one-year-long COVID persistence in HD patients.

## 1. Introduction

The ongoing global health crisis sparked by the COVID-19 pandemic has brought into focus various complications that arise after infection, often termed the long-term COVID-19 condition (long COVID) [1,2]. Long COVID manifests as a broad spectrum of symptoms persisting or emerging after the acute phase of the infection, impacting multiple organ systems and leading to enduring health issues [2]. The COVID-19 pandemic has underscored the critical importance of understanding the multifaceted impacts of the SARS-CoV-2 virus, particularly among populations with pre-existing health conditions [3]. Patients undergoing hemodialysis (HD) represent a uniquely vulnerable group due to their compromised immune systems, the necessity of frequent healthcare interactions, and the high prevalence of comorbid conditions such as cardiovascular diseases and diabetes. These factors not only elevate their risk of contracting COVID-19 but also predispose them to more severe outcomes, including the development of long COVID [4,5]. Despite the initial steps taken to explore the underlying mechanisms and potential predictors of long COVID within the general population [6,7,8], studies focusing on the HD population remain scarce.

Patients undergoing HD often experience nutritional deficiencies stemming from the nature of chronic kidney disease and dialysis treatment [9]. These deficiencies can have significant implications for the patient’s overall health and recovery, particularly in the context of COVID-19 [9]. The Controlling Nutritional Status (CONUT) score is a valuable tool for assessing the nutritional status of patients, utilizing easily obtainable clinical parameters such as serum albumin, total cholesterol levels, and lymphocyte count [10]. The CONUT score offers a comprehensive assessment by evaluating protein reserves, immune function, and caloric sufficiency, which are critical factors in the management of patients undergoing HD [11]. Its simplicity and non-invasive nature make it an ideal tool for routine use in clinical settings. Moreover, the CONUT score has been identified as a potential prognostic indicator for various outcomes in patients with COVID-19. Studies have suggested the CONUT score as a predictor of hospitalization duration, severity, risk of long COVID, and mortality in the general COVID-19 patient cohort [12,13,14]. However, its relevance to long COVID among HD patients remains unclear, highlighting a significant knowledge gap.

Oxidative stress, characterized by an imbalance between the production of reactive oxygen species and the body’s antioxidant defenses, has been implicated in the pathogenesis of long COVID [15,16]. Elevated levels of oxidative stress markers have been observed in patients with long COVID, indicating that oxidative stress may play a significant role in the persistence of symptoms and the overall intensity of the condition [16,17,18]. However, the precise relationship between oxidative stress and the persistence of long COVID symptoms in patients undergoing HD remains unclear. In this context, we hypothesized that malnutrition or suboptimal nutritional status may contribute to the prolonged oxidative damage observed in patients with long COVID, thereby exacerbating the severity and duration of symptoms. Therefore, the present study aimed to evaluate the association between pre-infection nutritional status, oxidative stress, and one-year-long COVID persistence in patients undergoing HD.

## 2. Materials and Methods

### 2.1. Study Design and Cohort Selection

This prospective observational cohort study was part of the “Mechanisms of Development and Therapeutic Targets of Post-COVID Syndrome in Dialysis Patients” project (National Study Registration Number 0122U000144) and was conducted at the State Institution “Institute of Nephrology of the National Academy of Medical Science of Ukraine” in Kyiv, between November 2021 and May 2023. The study protocol received approval from the Institute Ethics Committee (protocol number: 2-2021, dated 6 April 2021), and all participants provided written informed consent before inclusion. The research followed the principles outlined in the Declaration of Helsinki and other relevant ethical guidelines.

The study targeted patients aged 18 years or older who were undergoing HD treatments and had a confirmed history of COVID-19 infection. COVID-19 diagnosis was confirmed by either detecting SARS-CoV-2 RNA using real-time reverse transcription polymerase chain reaction (PCR) in nasopharyngeal swab specimens or observing imaging findings consistent with COVID-19. To ensure a diverse patient population, participants were recruited from three dialysis centers: the State Institution “Institute of Nephrology of the National Academy of Medical Science of Ukraine” in Kyiv, as well as two Dialysis Medical Centers operated by LLC “Nephrocenter” located in Kyiv and Zaporizhzhia, Ukraine. All enrolled patients had received at least 6 months of dialysis treatment before contracting COVID-19, maintained a stable clinical condition, and possessed a functioning arteriovenous fistula with a target Kt/V value of at least 1.2. The exclusion criteria comprised recent hospitalization, a history of cardiovascular events, undergoing immunosuppressive therapy, having systemic or malignant diseases, and experiencing acute inflammatory processes.

### 2.2. Sample Size

The sample size for our study was calculated using MedCalc version 19.2.6 (Ostend, Belgium) Statistical Software, based on a recent study by Zhao et al. In this study, Zhao et al. assessed the predictive value of the CONUT score in determining the risk of long COVID in a general patient cohort [13]. They reported long COVID incidence rates of 0.65% and 0.38% in high-CONUT and mild-CONUT groups, respectively (log-rank < 0.0001), with a total sample size of 151 patients. Based on these results, we determined that a minimum of 98 participants is needed to achieve a power of 0.95 and an alpha of 0.05. To accommodate potential dropouts and ensure adequate study power, we increased the sample size by 20% beyond the initial calculation, resulting in a total of 118 patients being included in the study.

### 2.3. Data Collection and Follow-Up

To categorize patients based on their pre-infection CONUT scores, we retrospectively recorded the latest available data before their COVID-19 contraction (on average 21 ± 13 days prior). Baseline data collection occurred three months after the onset of COVID-19, involving the gathering of demographic information, conducting routine clinical evaluations, and assessing oxidative stress markers. Predialysis blood samples of 10 milliliters were collected after an overnight fast and immediately processed. Subsequently, patients were monitored for over one year, with long COVID symptoms checked and recorded monthly during the follow-up period. The study endpoint was the persistence of long COVID symptoms throughout the one-year follow-up duration.

Long COVID diagnosis was established based on the persistence of at least one clinical symptom following COVID-19 infection, independent of other medical conditions. Symptoms indicative of long COVID in this study encompassed fatigue, dyspnea, insomnia, concentration or memory impairment, mood changes, chest and joint discomfort, palpitations, muscle pain, alterations in smell and taste, cough, headache, skin rash or hair loss, and diarrhea.

The severity of acute COVID-19 was classified into three tiers: asymptomatic (no apparent signs of COVID-19 with a positive PCR test), mild to moderate (displaying COVID-19 symptoms or pneumonia without requiring oxygen supply), and severe (necessitating hospitalization with oxygen assistance).

### 2.4. Routine Clinical Evaluations

The patient’s age, sex, duration of HD treatment, dialysis dose measured by single pool Kt/V level, anuria status, body mass index (BMI), comorbidities (such as diabetes mellitus, hypertension, and a history of cardiovascular disease), and COVID-19 vaccination status prior to contracting the virus were meticulously documented. BMI was calculated as weight in kilograms divided by the square of the height in meters. Anuria was defined as daily diuresis of less than 100 mL.

Biochemical and hematological parameters were measured using the automated Flexor Junior (Vital Scientific, Spankeren, The Netherlands) and the ABX Micros-60 (Horiba Medical, Montpellier, France) analyzers, respectively. Clinical laboratory parameters included blood levels of serum albumin, C-reactive protein (CRP), electrolytes, intact parathyroid hormone (PTH), total cholesterol, total lymphocyte count (TLC), and hemoglobin concentration. PTH levels were evaluated using an immunoradiometric assay, and electrolyte levels were determined through conventional autoanalyzer techniques.

### 2.5. CONUT Score Calculation

The CONUT score was assessed twice: once before and then 3 months after contracting COVID-19. It was determined by evaluating serum albumin level, total cholesterol concentration, and TLC. Each parameter was assigned individual scores: serum albumin (0, 2, 4, 6), total cholesterol concentration, and TLC (0, 1, 2, 3 for each). These scores were then categorized as normal, light, moderate, or severe, corresponding to ranges of 0–1, 2–4, 5–8, and 9–12, respectively, as detailed in Table 1 [10].

### 2.6. Oxidative Stress Markers Determination

The study assessed oxidative markers including malondialdehyde (MDA) in both serum (MDAs) and erythrocytes (MDAe) to gauge lipid peroxidation. Additionally, serum ceruloplasmin, transferrin, and sulfhydryl groups (SH groups) were measured to evaluate antioxidant defense [19]. In selecting antioxidant markers for our study, we aimed to choose markers that are both clinically accessible and relevant to oxidative stress and nutritional status. Ceruloplasmin was chosen due to its dual role as an acute-phase reactant and a copper-carrying protein with antioxidant properties, making it a pertinent marker for inflammation and oxidative stress, which are significant factors in the progression and persistence of long COVID symptoms [20]. Similarly, transferrin, which is involved in iron transport, can be indicative of nutritional status and has implications for COVID-19 severity due to its role in iron homeostasis [21,22]. Lastly, SH groups were included, as they reflect the thiol status of proteins, a direct measure of antioxidant capacity and redox balance, crucial for understanding the oxidative challenges faced by HD patients during and after COVID-19 infection [23].

For the measurement of MDAs and MDAe, blood samples were collected and separated into serum and erythrocyte components. In the serum samples (MDAs), 0.5 mL of serum was mixed with 1.5 mL of 0.025 M Tris buffer containing potassium chloride (pH 7.4) and incubated at 37 °C for 30 min. Following incubation, 1 mL of 17% trichloroacetic acid solution was added, and the samples were centrifuged. Then, 1 mL of 0.8% thiobarbituric acid solution was added to the supernatant of each sample tube, followed by boiling and subsequent measurement of absorbance at 532 nm. The concentration of MDA in each sample was calculated using appropriate standard curves and expressed as µmol/L.

Serum ceruloplasmin was determined by combining 0.05 mL of serum with 4 mL of 0.4 M acetic buffer solution (pH 5.5) and 0.5 mL of a 0.5% aqueous solution of 1,2-phenylenediamine dihydrochloride. The absorbance was measured at 530 nm, and the ceruloplasmin concentration was expressed in g/L.

Serum transferrin concentration was assessed by adding 0.2 mL of serum to 2 mL of a 0.2% solution of ammonium-iron(III)-citrate (pH 5.5–5.8). The absorbance was measured at 440 nm after one minute and after 30 min, and transferrin concentration was calculated as the difference between the absorbance readings, expressed in g/L.

The level of SH groups in serum was determined by dissolving 0.05 mL of serum in 0.5 mL of distilled water, followed by the addition of potassium iodide solution, starch solution, and phosphate buffer. Absorbance was measured before and after the application of iodine solution, and the concentration of SH groups was expressed as mmol/L.

Reagents used in the study were sourced as follows: tris(hydroxymethyl)aminomethane, tris(hydroxymethyl)aminomethane hydrochloride, malonaldehyde bis(diethyl acetal), 1,4-phenylenediamine dihydrochloride, human ceruloplasmin, sodium fluoride, potassium chloride, and potassium iodide from Sigma-Aldrich (Burlington, MA, USA), and trichloroacetic acid, thiobarbituric acid, ferric ammonium citrate, sodium hydrogen phosphate, and sodium acetate were procured from Merck (Darmstadt, Germany). Transferrin was obtained from BioChemica (Fluka, Darmstadt, Germany).

### 2.7. Statistical Analysis

MedCalc version 19.2.6 (Ostend, Belgium) statistical software was used. Descriptive statistics were reported as means with standard deviations (M ± SD) or medians with interquartile ranges [Me (Q1–Q3)] for continuous variables and as frequencies with percentages for categorical variables. The Shapiro–Wilk test was employed to assess the normality of data distribution.

Comparisons between the groups were performed using the Student’s *t*-test for normally distributed continuous variables and the Mann–Whitney U test for non-normally distributed continuous variables. Categorical variables were compared using the Chi-square test (χ^2^).

Partial correlation analysis was conducted to evaluate the association between pre-infection CONUT scores and oxidative stress markers, adjusting for post-COVID CONUT scores, age, sex, and HD duration. A multivariate logistic regression analysis was performed to determine variables independently associated with MDA concentration. Age, sex, HD duration, and all variables showing significant differences between the groups were included as potential confounders in the analysis.

The Kaplan–Meier survival analysis was employed to estimate the probability of long COVID persistence throughout the one-year follow-up period. Subsequently, to account for potential confounders, Cox proportional hazard regression models were utilized to investigate the association between pre-infection CONUT score and one-year-long COVID persistence. Initially, the analysis was conducted without adjustments. Then, adjustments were made for age, sex, HD duration, and all variables demonstrating significant differences between the groups (Model 1). Model 2 included adjustments from Model 1 and additionally incorporated MDA and ceruloplasmin concentrations.

## 3. Results

### 3.1. Baseline Patient Characteristics

**Figure 1:**
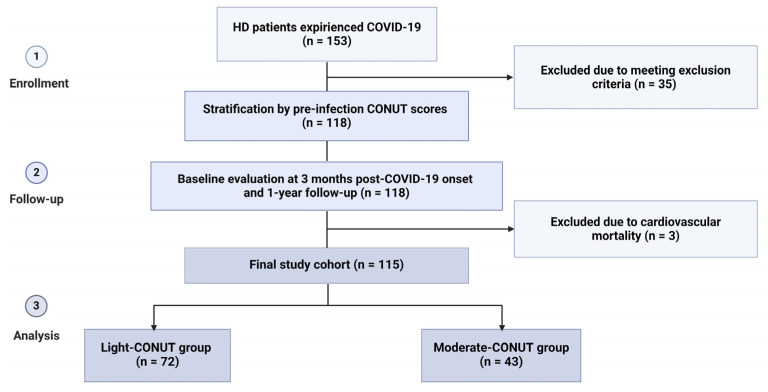
Study flowchart.

During the enrollment period, 153 patients undergoing HD who experienced COVID-19 were initially considered for inclusion in the study. Among them, 35 patients were excluded for various reasons, including a history of cardiovascular events (*n* = 9), short duration of dialysis before COVID-19 (*n* = 7), temporary vascular access (*n* = 7), recent hospitalization (*n* = 6), malignant or systemic disease (*n* = 5), and age less than 18 years (*n* = 1). Additionally, 3 out of the remaining 118 patients died due to heart failure during the follow-up period and were subsequently excluded from the study, resulting in a final study cohort of 115 patients (Figure 1).

**Figure 2:**
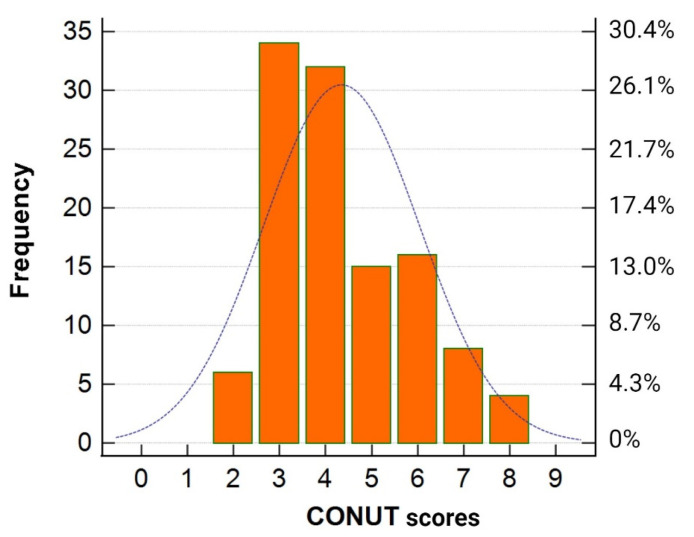
Distribution of the pre-infection CONUT scores in the study cohort.

The pre-infection CONUT scores ranged from 2 to 8 points (Figure 2), indicating that none of the patients in the study cohort were classified as having low or severe degrees of undernutrition before the onset of COVID-19.

Consequently, for further analysis, the patients were stratified into two groups: light CONUT and moderate CONUT.

As presented in Table 2, the patient characteristics, including age, sex, dialysis dose and vintage, prevalence of diabetes, arterial pressure, BMI, hemoglobin, electrolytes, and PTH levels, as well as medication used, were comparable between the light CONUT and moderate CONUT groups at three months post-COVID-19.

However, the moderate CONUT group demonstrated a higher proportion of anuric patients and lower levels of serum albumin, TLC, CRP, and cholesterol compared to the light CONUT group. Additionally, the moderate CONUT group had a lower prevalence of vaccinated patients and fewer cases of asymptomatic acute COVID-19. Although there was a tendency towards more hospitalizations with oxygen supply during the acute phase of COVID-19 in the moderate CONUT group, this difference did not achieve statistical significance. Moreover, the moderate CONUT group showed a significantly higher post-COVID CONUT score compared to the light CONUT group. Additionally, at three months post-COVID-19, severe undernutrition was present in nine (7.8%) patients across the cohort, with two (2.8%) in the light CONUT group and seven (16.3%) in the moderate CONUT group (χ^2^ = 74.9, *p* < 0.0001).

### 3.2. Pre-Infection CONUT Scores and Post-COVID Oxidative Damage

Analysis of oxidative stress markers indicated a significantly elevated concentration of MDAs and reduced ceruloplasmin level in the moderate CONUT group compared to the light CONUT group (Table 3).

In the partial correlation analysis, with post-COVID CONUT scores, age, sex, and HD duration as covariates, the pre-infection CONUT score showed a significant association with MDAs (r = 0.65, *p* < 0.0001) and ceruloplasmin levels (r = −0.27, *p* = 0.004).

**Figure 3:**
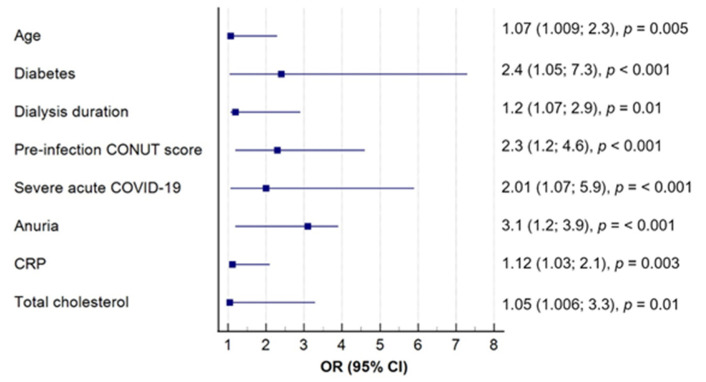
Predictors of oxidative damage in long COVID patients undergoing HD in the multivariate logistic regression analysis.

To further address potential confounding factors that could affect the intensity of oxidative stress, the patients were categorized based on median MDAs levels (<269 and ≥269 μmol/L). Subsequently, a multivariate logistic regression analysis was conducted to determine variables significantly affecting MDA concentration. Notably, the pre-infection CONUT score emerged as an independent predictor of post-COVID oxidative damage, independently of other factors (Figure 3).

### 3.3. Pre-Infection CONUT Scores and One-Year-Long COVID Persistence

**Figure 4:**
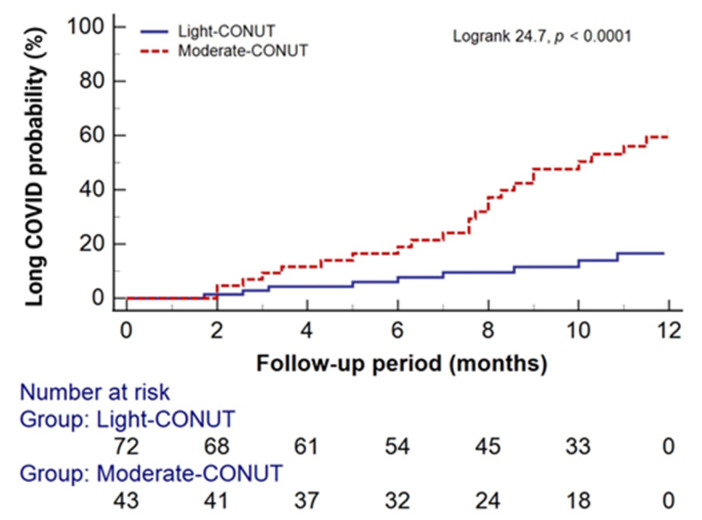
Kaplan–Meier curves of the probability of one-year-long COVID persistence stratified by the pre-infection CONUT scores.

Throughout the follow-up period, 83 (72.2%) patients receiving HD were diagnosed with long COVID. Among them, 41 (95.3%) belonged to the moderate CONUT group, and 42 (58.3%) were in the light CONUT group (χ^2^ = 22.1, *p* < 0.0001). At the end of the follow-up period, 48 (41.7%) patients still presented with long COVID, with 32 (74.4%) in the moderate CONUT group and 16 (22.2%) in the light CONUT group (χ^2^ = 29.9, *p* < 0.0001). Kaplan–Meier analysis revealed a significantly higher probability of one-year-long COVID persistence in the moderate CONUT group compared to the light CONUT group (Figure 4).

Cox proportional hazard models were utilized to assess the risk of one-year-long COVID persistence based on CONUT scores. As shown in Table 4, both unadjusted and adjusted models demonstrated a significantly higher risk for the one-year persistence of long COVID in the moderate CONUT group compared to the light CONUT group.

## 4. Discussion

The emergence of long COVID as a significant health concern post-acute SARS-CoV-2 infection has prompted heightened attention towards its predisposing factors [1,8,24]. Nutritional deficiency has been identified as a significant risk factor for severe acute COVID-19 and long COVID in the general patient population [12,13,25]. However, there is a lack of data on this topic in patients undergoing HD, who often have malnutrition at onset. To our knowledge, our study is the first to investigate the association between pre-infection nutritional status, measured by the CONUT score, post-COVID oxidative stress, and one-year-long COVID persistence. The findings revealed that patients with moderate pre-infection CONUT scores faced a heightened risk of severe undernutrition and increased oxidative stress three months post-acute COVID-19 compared to those with light CONUT scores. Moreover, moderate pre-infection undernutrition significantly contributed to oxidative damage and the persistence of long COVID symptoms at one year, independent of other known factors. It is worth noting that none of the patients in our cohort were classified as severely undernourished before COVID-19 infection, highlighting the importance of even mild to moderate nutritional deficiencies in affecting long-term COVID-19 outcomes.

Although data on the HD population are limited, our findings align with broader research suggesting that malnutrition can worsen the severity of COVID-19 and contribute to prolonged recovery times in the general patient population [14,26,27,28,29]. Malnutrition, characterized by deficiencies in essential nutrients, compromises immune function and increases vulnerability to infections [27,28,30]. This is particularly relevant for patients undergoing HD, who are already at an increased risk for malnutrition due to factors such as reduced dietary intake, nutrient losses during dialysis, and chronic inflammation [9,31,32]. Consistent with our results, Lin et al. have emphasized the notable impairment in humoral response to COVID-19 vaccination among HD patients identified as malnourished based on the CONUT score [32]. This corresponds with our observation that moderate pre-infection CONUT score status predisposes HD patients to prolonged long COVID symptoms, even among those who have been vaccinated.

Furthermore, our study highlights the role of oxidative stress in the development and persistence of long COVID symptoms. Oxidative stress has been implicated in the pathogenesis of long COVID, as evidenced by previous research [16,18,33]. Elevated levels of oxidative damage markers, such as MDAs, and reduced levels of ceruloplasmin were observed in our cohort of HD patients with moderate pre-infection nutritional deficiencies, suggesting that oxidative stress may play a significant role in the chronicity of COVID-19 sequelae. Our previous report also demonstrated significantly increased oxidative stress and decreased antioxidant markers in patients undergoing HD with long COVID compared to fully recovered patients [34]. Additionally, in line with our findings, Stufano et al. proposed MDAs as an independent predictor of long COVID in Italian workers [35], and Restea et al. demonstrated decreased ceruloplasmin levels in patients with type 2 diabetes and severe COVID-19 [36].

The association between the CONUT score and oxidative stress biomarkers is multifaceted. Serum albumin, one of the components of the CONUT score, is a well-known negative acute-phase reactant, which means its levels decrease in response to systemic inflammation and oxidative stress [37,38]. This decrease can be attributed to the increased catabolism and reduced synthesis of albumin during oxidative stress [38]. Additionally, albumin itself has antioxidant properties, as it can bind and neutralize a variety of oxidants [37,38]. Therefore, low serum albumin levels, as indicated by a higher CONUT score, may reflect a compromised antioxidant defense system. Total cholesterol, another parameter of the CONUT score, is also involved in the body’s antioxidant network. Cholesterol is a component of cell membranes and serves as a precursor for steroid hormones and bile acids [37]. Oxidative stress can lead to the peroxidation of lipids, including cholesterol, which disrupts cellular membranes and signaling pathways, further exacerbating the oxidative damage [37,39]. Thus, alterations in total cholesterol levels could signal disruptions in the body’s ability to maintain redox homeostasis and protect against oxidative stress. Lymphocyte count, the third component of the CONUT score, is indicative of the immune system’s status [37,38]. Lymphocytes play a crucial role in the immune response and are sensitive to oxidative stress, which can impair their function and lead to immunosuppression [37,40]. A low lymphocyte count may suggest a weakened immune system, which is less capable of combating oxidative stress and inflammation that can further deteriorate nutritional status.

The interplay between nutritional status and oxidative stress is further complicated by the fact that oxidative stress can lead to malnutrition by impairing appetite and increasing catabolism [31,41]. In the context of long COVID, this cycle can be particularly detrimental. Nutritional deficiencies can lead to diminished antioxidant defenses, thereby increasing vulnerability to oxidative damage [28,42,43,44]. Conversely, oxidative stress can further deplete nutrients, creating a vicious cycle that can be particularly detrimental in the context of long COVID [33,45]. For example, the depletion of antioxidants like glutathione, superoxide dismutase, and catalase has been linked to severe COVID-19 outcomes and may contribute to the persistence of long COVID symptoms [46,47].

Our study supports the hypothesis that optimizing nutritional status could mitigate oxidative stress levels, potentially reducing the severity and duration of long COVID symptoms in vulnerable populations like those undergoing HD. To translate the study findings into clinical practice, we advocate for the integration of routine nutritional assessments, such as the CONUT score, into patient care protocols. This tool allows for an assessment of nutritional status and the identification of patients at risk of long COVID outcomes. Once identified, targeted interventions, including dietary counseling, oral nutritional supplements, and, if necessary, parenteral nutrition support, can be initiated to address specific nutritional deficiencies. Furthermore, interdisciplinary collaboration between nephrologists, dietitians, and other healthcare professionals is essential for developing comprehensive nutritional care plans tailored to the individual needs of patients undergoing HD. This collaborative approach allows for the integration of dietary interventions, pharmacological therapies, and lifestyle modifications to optimize nutritional status and oxidative balance, mitigating the risk of severe acute COVID-19 and long COVID symptoms.

Despite the novel insights provided by our study, several limitations warrant consideration. First, the observational nature of our study precludes causal inference, and unidentified confounding factors may influence the observed associations. Second, the relatively small sample size limits the generalizability of our findings. Third, the exclusion criteria, such as excluding patients with recent hospitalization or cardiovascular events, may introduce selection bias and limit the representativeness of the study cohort. Fourth, though ceruloplasmin, transferrin, and SH groups were used in this study as markers of antioxidant capacity, they may not fully reflect the overall redox state. A more comprehensive assessment of oxidative stress might include markers such as glutathione, oxidized glutathione, carbonylated proteins, and DNA damage measured with 8-oxoguanine, offering a more detailed evaluation of the balance between oxidative and reductive processes. Next, variability in the measurement of oxidative stress markers and other variables could affect the accuracy and reliability of the results. Finally, although efforts were made to control for the potential confounders, residual confounding may still exist. Factors such as age, comorbidities, medication use, dietary habits, socioeconomic status, and variations in hemodialysis treatment protocols could have confounded the observed associations between pre-infection nutritional status, oxidative stress, and the persistence of long COVID symptoms.

Moving forward, additional research is warranted to delve deeper into the mechanisms underlying the interplay between nutritional status, oxidative stress, and long COVID persistence in patients undergoing HD. Prospective studies with larger and more diverse cohorts could provide further insights into the causal relationships and potential therapeutic targets in mitigating long COVID symptoms in this vulnerable population. Additionally, interventional studies evaluating the efficacy of targeted nutritional interventions in improving long-term COVID-19 outcomes among HD patients are warranted. These include dietary modifications and antioxidant supplementation. Moreover, exploring the impact of socioeconomic factors, dietary habits, and variations in HD treatment protocols on the observed associations could enhance our understanding of the complex interactions involved in long COVID pathogenesis and persistence in this patient population.

## 5. Conclusions

Together, our study highlights the association between pre-infection nutritional status, oxidative stress, and the one-year persistence of long COVID in patients undergoing HD. Patients with moderate pre-infection nutritional deficiencies, as assessed by the CONUT score, faced a heightened risk of severe undernutrition following acute COVID-19, increased oxidative stress, and prolonged long COVID symptoms compared to those with light nutritional deficiencies. The findings underscore the importance of addressing even mild to moderate nutritional deficiencies in improving long COVID outcomes among HD patients. Strategies aimed at optimizing nutritional status may help mitigate oxidative stress levels and reduce the severity and duration of long COVID symptoms in this vulnerable population. 

Further research, including larger prospective studies with diverse cohorts, is needed to validate these findings and elucidate the underlying mechanisms linking nutritional status, oxidative stress, and long COVID persistence in HD patients. Additionally, interventional studies evaluating the efficacy of nutritional interventions in mitigating long COVID symptoms in this population are warranted.

## Figures and Tables

**Table 1:** CONUT score calculation.

Parameter	Undernutrition Degree			
	Normal	Light	Moderate	Severe
Serum albumin, g/L	30.5–40.5	30.0–30.49	20.5–20.9	<20.5
Score	0	2	4	6
TLC, /mm^3^	>1600	1200–1599	800–1199	<800
Score	0	1	2	3
Cholesterol, mmol/L	>4.65	3.62–4.65	2.59–3.61	<2.59
Score	0	1	2	3
Screening total score	0–1	2–4	5–8	9–12

Abbreviations: TLC, total lymphocyte count.

**Table 2:** Baseline characteristics of the study participants stratified by the pre-infection CONUT scores.

Clinical Parameters	All Patients (n = 115)	Light CONUT Group (n = 72)	Moderate CONUT Group (n = 43)	*p*-Value
Demographic and clinical data				
Male sex, n (%)	50 (43.5%)	34 (47.2%)	16 (37.2%)	0.29
Age, years	52.2 ± 11.6	49.8 ± 10.6	53.7 ± 12.1	0.08
Diabetes	17 (14.8%)	10 (13.9%)	7 (16.3%)	0.73
Systolic blood pressure, mm Hg	130 (120–140)	130 (120–140)	130 (130–140)	0.14
Diastolic blood pressure, mm Hg	80 (70–90)	80 (70–85)	80 (72–90)	0.32
BMI, kg/m^2^	25.0 (22.1–30.1)	25.3 (22.4–30.3)	24.3 (21.1–29.4)	0.36
Time on HD, months	72.0 (30.3–138.2)	69.0 (29.5–126.0)	72.0 (36.0–163.7)	0.36
spKt/V	1.4 (1.3–1.6)	1.4 (1.3–1.7)	1.3 (1.2–1.6)	0.16
Anuric patients, n (%)	58 (50.4%)	31 (31.9%)	27 (62.8%)	0.001
Serum albumin, g/L	35.2 (34.2–40.6)	36.5 (34.1–41.6)	34.7 (31.5–38.7)	0.003
TLC, mm^3^	1200 (824–3760)	1200 (780–3750)	981 (560–2350)	0.04
CRP, mg/L	5.9 (4.6–10.7)	4.6 (3.3–9.4)	6.8 (5.1–11.2)	0.02
Hb, g/L	102 (95–108)	103 (95–107.5)	102 (95–108)	0.65
Potassium, mmol/L	4.42 (3.9–5.1)	4.7 (3.9–5.7)	4.3 (3.8–4.9)	0.76
Calcium, mmol/L	2.25 (2.1–2.4)	2.28 (2.1–2.4)	2.16 (2.1–2.3)	0.62
Phosphorus, mmol/L	1.75 (1.5–2.1)	1.6 (1.4–1.9)	1.8 (1.5–2.1)	0.14
PTH, ng/L	259 (164–381)	222.3 (125.2–352.6)	303.6 (192.5–476.8)	0.06
Total cholesterol, mmol/L	4.9 ± 1.18	5.2 ± 1.17	4.5 ± 1.07	0.002
CONUT score	4 (4–6)	4 (3–4)	6 (5–7)	<0.0001
Medications				
ACE inhibitors/RAAS blockers, n (%)	64 (5.6%)	42 (58.3%)	22 (51.2%)	0.46
Beta blockers, n (%)	81 (70.4%)	55 (76.4%)	26 (60.4%)	0.07
Calcium channel blockers, n (%)	76 (66.1%)	49 (68.1%)	27 (62.8%)	0.56
Alpha blockers, n (%)	19 (16.5%)	11 (15.3%)	8 (18.6%)	0.64
Iron supplementation, n (%)	63 (54.8%)	39 (54.2%)	24 (55.8%)	0.87
Erythropoietins, n (%)	96 (83.5%)	61 (84.7%)	35 (81.4%)	0.65
Non-calcium phosphate binders, n (%)	45 (39.1%)	28 (38.9%)	17 (39.5%)	0.95
Vaccination status and acute COVID-19 severity				
Vaccinated status for COVID-19, n (%)	40 (34.8%)	32 (44.4%)	8 (18.8%)	0.005
Asymptomatic COVID-19, n (%)	24 (20.9%)	21 (29.2%)	5 (11.6%)	0.03
Mild to moderate COVID-19, n (%)	73 (63.5%)	41 (56.9%)	26 (60.5%)	0.71
Hospitalization with oxygen supply, n (%)	18 (15.6%)	10 (13.9%)	12 (27.9%)	0.07

The values are expressed as exact numbers and proportions, mean ± standard deviation (M ± SD), or median (Me) with (interquartile range, Q25–Q75) and compared between the groups using the Chi-square test, Student’s *t*-test, and Mann–Whitney U test, as appropriate. Abbreviations: ACE, angiotensin-converting enzyme; BMI, body mass index; CONUT, controlling nutritional status; CRP, C-reactive protein; Hb, hemoglobin; PTH, intact parathyroid hormone; RAAS, renin–angiotensin–aldosterone system; spKt/V, single-pool Kt/V; TCL, total lymphocyte count.

**Table 3:** Comparison of oxidant-antioxidant markers in patients undergoing HD stratified by the pre-infection CONUT scores.

Markers	All Patients (n = 115)	Light CONUT Group (n = 72)	Moderate CONUT Group (n = 43)	*p*-Value
MDAs, μmol/L	269.0 (218.0–368.7)	240.3 (205.1–274.5)	403.6 (258.2–512.7)	<0.0001
MDAe, μmol/L	769.0 (641.3–945.5)	750.4 (728.1–936.3)	859.3 (746.4–948.8)	0.051
Transferrin, g/L	2.5 (1.7–5.0)	3.0 (1.7–5.2)	2.3 (1.6–5.1)	0.49
Ceruloplasmin, g/L	0.19 (0.12–0.24)	0.21 (0.14–0.31)	0.14 (0.11–0.21)	0.0009
SH groups, mmol/L	1.56 (1.34–1.76)	1.56 (1.42–1.75)	1.54 (1.34–1.81)	0.89

The values are expressed as median (Me) and (interquartile range, Q25–Q75) and compared between the groups using the Mann–Whitney U test. Abbreviations: CONUT, controlling nutritional status; MDAs, serum malondialdehyde; MDAe, erythrocytes malondialdehyde;- SH groups, sulfhydryl groups.

**Table 4:** Association between pre-infection CONUT score and one-year persistence of long COVID.

Variable	b	SE	Wald χ^2^	*p*-Values	HR (95% CI)
Unadjusted	1.3	0.31	18.4	<0.0001	3.7 (2.1; 6.8)
Model 1	1.9	0.38	4.85	<0.0001	6.8 (3.2; 9.4)
Model 2	1.5	0.61	6.5	0.01	4.6 (1.4; 9.9)

Abbreviations: b, coefficient estimates; CI, confidence interval; HR, hazard ratio; SE, standard error. Model 1 was adjusted for age, sex, dialysis duration, diabetes, anuria, vaccination status, TLC, serum albumin, CRP, and cholesterol levels. Model 2 was additionally adjusted for MDA and ceruloplasmin concentrations.

## Data Availability

The data used in the study are available upon reasonable request to the corresponding author.
